# Border row effects improved the spatial distributions of maize and peanut roots in an intercropping system, associated with improved yield

**DOI:** 10.3389/fpls.2024.1414844

**Published:** 2024-06-26

**Authors:** Qiqi Dong, Xinhua Zhao, Yuexin Sun, Dongying Zhou, Guohu Lan, Junyu Pu, Chen Feng, He Zhang, Xiaolong Shi, Xibo Liu, Jing Zhang, Zhanxiang Sun, Haiqiu Yu

**Affiliations:** ^1^ College of Agronomy, Shenyang Agricultural University, Shenyang, Liaoning, China; ^2^ Tillage and Cultivation Research Institute, Liaoning Academy of Agricultural Sciences, Shenyang, Liaoning, China; ^3^ National Agricultural Experimental Station for Agricultural Environment, Fuxin, Liaoning, China; ^4^ College English Department, Shenyang Agricultural University, Shenyang, Liaoning, China; ^5^ School of Agriculture and Horticulture, Liaoning Agricultural Vocational and Technical College, Yingkou, Liaoning, China

**Keywords:** maize-peanut intercropping, interspecific interaction, root length density, root surface area density, special root length, root diameter

## Abstract

**Background:**

Border row effects impact the ecosystem functions of intercropping systems, with high direct interactions between neighboring row crops in light, water, and nutrients. However, previous studies have mostly focused on aboveground, whereas the effects of intercropping on the spatial distribution of the root system are poorly understood. Field experiments and planting box experiments were combined to explore the yield, dry matter accumulation, and spatial distribution of root morphological indexes, such as root length density (RLD), root surface area density (RSAD), specific root length (SRL), and root diameter (RD), of maize and peanut and interspecific interactions at different soil depths in an intercropping system.

**Results:**

In the field experiments, the yield of intercropped maize significantly increased by 33.45%; however, the yield of intercropped peanut significantly decreased by 13.40%. The land equivalent ratio (LER) of the maize–peanut intercropping system was greater than 1, and the advantage of intercropping was significant. Maize was highly competitive (*A* = 0.94, CR=1.54), and the yield advantage is mainly attributed to maize. Intercropped maize had higher RLD, RSAD, and SRL than sole maize, and intercropped peanut had lower RLD, RSAD, and SRL than sole peanut. In the interspecific interaction zone, the increase in RLD, RSAD, SRL, and RD of intercropped maize was greater than that of intercropped peanut, and maize showed greater root morphological plasticity than peanut. A random forest model determined that RSAD significantly impacted yield at 15–60 cm, while SRL had a significant impact at 30–60 cm. Structural equation modeling revealed that root morphology indicators had a greater effect on yield at 30–45 cm, with interactions between indicators being more pronounced at this depth.

**Conclusion:**

These results show that border-row effects mediate the plasticity of root morphology, which could enhance resource use and increase productivity. Therefore, selecting optimal intercropping species and developing sustainable intercropping production systems is of great significance.

## 1 Introduction

Intercropping is a sustainable and intensive cropping pattern that has the advantages of improving crop yields (Liu et al., 2020; Zou et al., 2021) and land use efficiency (Yu et al., 2015; Feng et al., 2021), inhibiting weeds, and reducing pests and diseases (Brooker et al., 2015; Beillouin et al., 2021; Chadfield et al., 2022). Many previous studies have confirmed that intercropping of gramineous and leguminous crops has yield advantages, for instance, intercropping of maize and soybean, maize and peanut (Li et al., 2019), and sorghum and soybean (Wang et al., 2021a). Studies have shown that the “solar corridor crop system” or other intercropping designs can open up light access to lower portions of the maize canopy, increasing light interception and light energy efficiency in intercropped maize (Kremer and Deichman, 2014a). When this design is used with peanuts as the intercrop, the content and conversion efficiency of precursors for chlorophyll synthesis are also increased (Lu et al., 2023), thereby improving canopy light energy efficiency and promoting increased yield (Nelson, 2014; Wang et al., 2020; Wang et al., 2021b). In addition, soil quality in this system was improved through increased organic carbon and nitrogen content, efficient nutrient cycling, and increased microbial activity (Kremer and Deichman, 2014b). It was evident that the existence of competitive and complementary roles among intercrops is one of the important factors influencing yield advantage. These are among the reasons why intercropping results in a yield advantage, with the aboveground and underground parts affecting the productivity.

Based on complementary effects, combinations of deep-rooted and shallow-rooted crops are used to obtain soil resources at different depths and alleviate underground competition, which enable sufficient resource use and growth by both species (Hassan et al., 2019; Oburger et al., 2022). It was demonstrated that, in the intercropping system of proso millet and mung bean, the root length density, root surface area density, and root volume density of the dominant crop, proso millet, increased in the top soil layer, which helped to absorb more soil moisture and enhance the water use efficiency of proso millet (Gong et al., 2020). Compared with legumes, intercropped maize had more plasticity in root morphology, with greater variation in root length density, root weight density, and total root surface area. Maize’s root system occupied more soil space in the intercropping, which inhibited the lateral distribution of root length density in the legume crop (Yang et al., 2022). The maize/soybean strip intercropping system promoted root growth and distribution in both crops, resulting in 72.15% and 15.72% increase in root length density in maize and soybean, respectively, as well as improved soil water use and productivity (Te et al., 2023).

Our previous study showed that the maize and peanut root interactions improved soil nitrogen utilization, exhibiting a nitrogen utilization advantage for side-row maize (Dong et al., 2022; Zhao et al., 2022a). Side-row maize was larger than the middle-row ones in terms of both above-ground partial light competition and below-ground nitrogen utilization, showing a border-row effects, for example, in a wheat–maize strip intercropping system, the absorbed photosynthetically active radiation of side-row wheat was higher than that of middle-row wheat, and side-row maize did not show an advantage in absorbing photosynthetically active radiation due to interspecific competition (Wang et al., 2017). In the maize–soybean strip intercropping system, the root system of maize extended into the side rows of the soybean rows and was concentrated in the 0–20-cm soil layer. Intercropping also increased the soil moisture and nitrate content of the side-row maize in the 20–60 cm soil layer (Shen et al., 2023). Previous studies have focused on the border-row effect on root distribution, soil nutrient, and water use. However, there are fewer studies on the effects of border-row effect on the spatial distribution characteristics of maize and peanut roots and on exploring the potential effects on crop yields.

We hypothesized that differences in root morphological characteristics and the spatial distribution of roots caused by border-row effects influenced productivity in maize–peanut intercropping systems. This study was conducted with field experiments and planting box experiments to explore the differences in yield, dry matter accumulation, root morphology, and interspecific interactions between maize and peanut under the three planting patterns of sole maize, sole peanut, and maize and peanut intercropping. The main purposes are to (1) alter the maize and peanut yields under sole and intercropping planting patterns, (2) determine the differences in the root characteristics and spatial distribution of maize and peanut under sole and intercropping systems, and (3) quantify the correlation between interspecific interactions and root morphological indicators of maize and peanut in intercropping systems. Additionally, this investigation sought to analyze the plasticity of root morphology and spatial distribution in maize–peanut intercropping systems from the point of view of competition and facilitation. The results highlight the border-row effects on the spatial distribution and productivity of the root system of each crop in the intercropping system, which is important for the improvement and promotion of maize–peanut intercropping systems in the future.

## 2 Materials and methods

### 2.1 Experimental site

This experiment was carried out in the experimental field of Shenyang Agricultural University at the Crop Cultivation Science Observation Station in Northeast China of the Ministry of Agriculture and Rural Areas (41°82′ N, 123°56′ E) from 2020 to 2021. The monthly average temperature was 19.75°C, and the monthly average precipitation was 120.69 mm in the growing season (**Supplementary Figure S1**). The soil was brown loam, which was classified according to the Chinese Soil Taxonomy. Before the 2020 experiment, the soil contained 14.59 g·kg^-1^ of organic matter, 178.07 mg·kg^-1^ of available nitrogen, 43.82 mg·kg^-1^ of available phosphorus, and 201.86 mg·kg^-1^ of available potassium, with a pH of 6.5. Before the 2021 experiment, the soil contained 14.85 g·kg^-1^ of organic matter, 199.64 mg·kg^-1^ of available nitrogen, 56.87 mg·kg^-1^ of available phosphorus, and 213.36 mg·kg^-1^ of available potassium, with a pH of 6.5.

### 2.2 Experimental design

**Figure 1:**
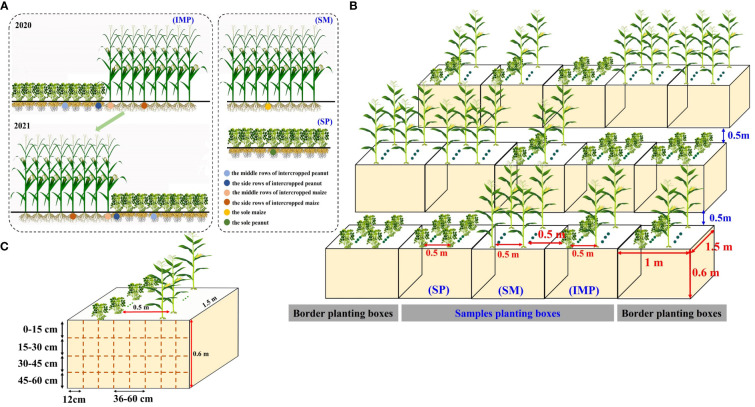
Schematic diagram of the planting pattern. **(A)** Row configuration and sampling positions for the field experiment. **(B)** Row configuration for the planting box experiment. **(C)** Sampling positions for the planting box experiment. SM, sole maize; SP, sole peanut; IMP, intercropping of maize and peanut. 0–36 cm: peanut inner row, 36–60 cm: interaction zone, 60–96 cm: maize inner row.

A single-factor randomized block design was used with three planting patterns: sole maize (SM), sole peanut (SP), and an 8:8 rotational strip intercropping of maize and peanut (IMP), with three replications (**Figure 1A**). Intercropped maize strips and intercropped peanut strips were rotated interannually from 2020 to 2021 to prevent issues related to continuous peanut cropping and maintain soil fertility. The row spacing in the sole maize and intercropped maize strips was 50 cm, the plant spacing was 25 cm, and the plant density was 66,670 plants·hm^-2^. The row spacing in the sole peanut and intercropped peanut strips was 50 cm, the plant spacing was 12.3 cm, and the density was 271,016 plants·hm^-2^. Planting proceeded from north to south, covering a length of 10 m. The width of the maize–peanut intercropped strips was 3.5 m, and the band width was 8.0 m. There were 24 rows with only maize and only peanut. The area of the maize and peanut only plots was 120 m^2^, and the area of the intercropping plots was 80 m^2^.

To better analyze the changes in root spatial distribution at different soil depths, a planting box experiment was carried out (**Figure 1B**) with three planting patterns (three repetitions): SM, SP, and IMP. To replicate the field conditions and reduce damage to the field soil during destructive harvesting, this experiment was established near the field experiment. Although our planting box experiment may lead to certain limitations (reduced shading of peanuts by maize compared to the field experiment), we ensured consistency with the field experiment, e.g., (1) ensured that the planting direction (north–south) of maize and peanuts was the same as what actually occurred in the field experiment and (2) the planting boxes for each treatment were closely spaced, but the aisle distance between each replication was 0.5 m (**Figure 1B**). Soil was collected from the 0–20 cm soil layer in the long-term field experiment. After the soil was dried, it was filtered through a 2-mm sieve, and 1,000 kg of soil was placed in each box. One row of maize and one row of peanut were planted in the intercropping box, with a row spacing of 50 cm, maize plant spacing of 25 cm, and peanut plant spacing of 6 cm. The planting boxes with sole maize and sole peanut had two rows of maize and two rows of peanut, respectively, and the row spacing and plant spacing were the same as those in the intercropping system. The amounts of fertilizer applied in the field experiment and planting box experiment are shown in **Supplementary Table S1**. Other cultivation and management practices were the same as those in conventional field production.

The maize variety was the hybrid Liangyu 99, which was supplied by Dandong Denghai Liangyu Seed Industry Co., Ltd., and the peanut variety was Nonghua 9, which was supplied by the Peanut Research Institute of Agricultural College of Shenyang Agricultural University. Maize and peanut were sown on May 15, 2020 and May 18, 2021 and harvested on September 19, 2020 and September 18, 2021, respectively.

### 2.3 Sampling

#### 2.3.1 Yield and dry matter accumulation

The yields of maize and peanut were measured at the harvest stage in the 2020–2021 field experiments. In the plots with only maize and only peanut, all the plants in the 2 m long maize or peanut strip within each row for four consecutive rows were harvested. In the maize–peanut intercropped plots, all the plants in the 2 m long maize or peanut strip within each row for four consecutive rows were harvested. The grains were sun-dried and weighed after threshing by hand.

At the harvest stage, three maize plants and three peanut plants were collected in intercropped maize (IM), the middle row of intercropped maize (MIM), intercropped peanut (IP), the middle row of intercropped peanut (MIP), SM, and SP in the field experiments (**Figure 1A**), and three repetitions of this collection were performed. In the planting box experiment, one maize plant and three peanut plants were collected in IM, IP, SM, and SP, with three repetitions (**Figure 1B**). The roots, stems, leaves, and grain/pod parts were separated and dried at 105°C for 30 min and 80°C to constant weight for dry matter accumulation.

### 2.4 Spatial distribution of root morphology

In the flowering and needle setting stage of peanut in 2020–2021, root sampling was conducted for IMP, SM, and SP of the planting box experiment (with three repetitions) to determine the root spatial distribution. A long-bladed knife and an iron plate were used to cut the soil into blocks. The soil samples were taken in layers of 15 cm each and at a volume of 20 × 12 × 15 cm^3^ (**Figure 1C**). The soil samples were placed in mesh bags and washed with water until the roots could be clearly identified. The roots of maize and peanut can be distinguished by color, texture, and branching pattern. Maize roots are white and smooth, while those of peanuts are brown with nodules. The root samples were scanned with an EpsonPerfection V700 root scanner and then analyzed with WinRHIZO root analysis software to determine the root length, root surface area, and average root diameter. Finally, the roots were dried at 80°C in an oven and weighed.

### 2.5 Calculations

#### 2.5.1 Land equivalent ratio

The land equivalent ratio (LER) was used to assess the advantage of intercropping systems (Ma et al., 2019). LER was calculated as follows:


(1) $$\text{LER}=\frac{\text{Yim}\times \text{Pm}}{\text{Ysm}}+\frac{\text{Yip}\times \text{Pp}}{\text{Ysp}}$$

where Yim and Ysm are the yields of (kg hm^-2^) intercropped maize and sole maize, respectively. Yip and Ysp are the yields of (kg hm^-2^) intercropped peanut and sole peanut, respectively. Pm and Pp are the sown proportion of maize and peanut in the intercropping system (Pm = 1/2 and Pp = 1/2), respectively. LER greater than 1.0 indicates interspecific stimulation and a land use advantage from intercropping. Conversely, when LER is less than 1.0, there is no advantage from intercropping.

#### 2.5.2 Interspecies interaction index

Aggressivity (*A*) is an index that measures interspecies competition in intercropping systems by comparing the yields of intercropping and single cropping as well as their respective land occupancies (Dhima et al., 2007; Yin et al., 2019).


(2) $$A=\frac{\text{Yim}}{\text{Ysm}\times \text{Pm}}-\frac{\text{Yip}}{\text{Ysp}\times \text{Pp}}$$

where *A* is the aggressiveness of maize relative to peanut in the intercropping systems. If *A* is greater than 0, the competitiveness of maize is greater than that of peanut in the intercropping system; otherwise, maize is less competitive.

The system productivity (kg hm^-2^) represents the productivity of the entire intercropping system (Gong et al., 2020).


(3) System productivity=Yim×Pm+Yip×Pp

The competitive ratio (CR) is another indicator used to assess the competitiveness of different species in intercropping systems (Liu et al., 2015).


(4) $$\text{CR}=\frac{\text{Yim}}{\text{Ysm}\times \text{Pm}}/\frac{\text{Yip}}{Y\text{sp}\times \text{Pp}}$$

where CR is the competitiveness of maize relative to peanut. If CR is greater than 1.0, the competitiveness of maize is higher than that of peanut in the intercropping system. Otherwise, the competitiveness is lower than that of peanut.

#### 2.5.3 Root length density

Root length density (RLD) is the root length per unit soil volume (cm cm^–3^), which was calculated with the following formula (Zhang et al., 2011):


(5) $$\text{RLD}=\frac{L}{V}\;$$

where *L* is the root length (cm) and *V* is the volume of the soil sample (3,600 cm^3^).

#### 2.5.4 Root surface area density

Root surface area density (RSAD) is the root surface area per unit soil volume (cm^2^ cm^–3^), which was calculated with the following formula (Duan et al., 2017):


(6) $$\text{RSAD}=\frac{S}{V}\;$$

where *S* is the root surface area (cm^2^) and *V* is the volume of the soil sample (3600 cm^3^).

#### 2.5.6 Specific root length

SRL is the ratio of root length to root weight and shows the relationship between root penetration intensity and underground biomass allocation (Wang et al., 2014).


(7) $$\text{SRL}=\frac{L}{\text{DW}}\;$$

where *L* is the root length (cm) and DW is the root weight of the soil sample.

### 2.6 Statistical analysis

The yield, dry matter accumulation, and interspecific interaction index in field experiments were assessed by one-way analysis of variance (ANOVA) with Duncan’s test, and maize and peanut dry matter accumulation in the planting box experiment were assessed by Student’s *t*-test using SPSS 26.00 (IBM SPSS Inc., NY, USA). Differences were considered statistically significant at *P*< 0.05. Origin 2023 (Origin Lab Corporation, Northampton, MA, United States) was used to map root length density, root surface area density, specific root length, and root diameter. The yield per plant and the root morphological indexes of maize and peanut were normalized by principal component analysis (PCA). Pearson correlation analysis was performed to examine the potential relationship between the maize and peanut root morphological indexes and the yield per plant of maize and peanut. The random forest model evaluated the significant predictors affecting yield per plant, including root length density, root surface area density, specific root length, and root diameter. These analyses were conducted using the random forest software package. The model significance and predictor importance were verified using the rfUtilities and rfPermute packages in the R software, respectively (Ju et al., 2023; Yuan et al., 2023). Structural equation modeling (SEM) was conducted using the R “piecewiseSEM” package to evaluate the direct and indirect relationships among yield per plant, root length density, root surface area density, specific root length, and root diameter (Jing et al., 2023; Zhao et al., 2023b).

## 3 Results

### 3.1 Changes in maize and peanut yields, interspecific interactions, and dry matter accumulation in field experiments

In the field experiments, compared with that of SM, the yield of IM significantly increased by 33.45% (2-year average, **Table 1**). Compared with that of SP, the yield of intercropped peanut significantly decreased by 13.40% (2-year average, **Table 1**). In 2020–2021, LER exceeded 1, indicating that intercropping of maize and peanut had advantages and improved the utilization of land resources (**Table 1**). On average, the 2-year productivity of the system was 8,748.34 kg hm^-2^. In addition, *A* exceeded 1, and a significant effect was observed between years (**Table 1**), indicating that maize was a dominant crop and had a greater competitiveness in the intercropping system. This result was supported by the CR, with a 2-year average greater than 1, and maize had a greater competitiveness than peanuts (**Table 1**).

**Table 1:** Changes in maize and peanut yields and interspecies interaction in field experiments.

Planting patterns	2020	2021	2-year average
SM	10,631.39 ± 4.11	10,756.94 ± 4.11	10,694.17 ± 0.42
IM	14,555.56 ± 5.85**	13,986.39 ± 4.19**	14,270.97 ± 5.02
SP	3,796.44 ± 5.67**	3,652.78 ± 0.25**	3,724.61 ± 2.92
IP	3,305.56 ± 1.34	3,145.83 ± 1.67	3,225.69 ± 0.72
LER	1.12	1.08	1.1
System productivity (kg hm^-2^)	8,930.56	8,566.11	8,748.34
Aggressivity (A)	1	0.88	0.94
Competitive ratio (CR)	1.57	1.51	1.54

* and ** indicate significant differences between different planting patterns at *P*< 0.05 and *P*< 0.01, respectively.

SM, sole maize; SP, sole peanut; IM, intercropped maize; IP, intercropped peanut. LER, Land equivalent ratio; based on Equation (1); A, Aggressivity; based on Equation (2); System productivity based on Equation (3); CR, Competitive ratio; based on Equation (4).

In the field experiment, the changes in dry matter accumulation per plant of maize and peanut order was IM > MIM > SM, SP > MIP > IP (**Supplementary Figure S2**). Compared with those of SM, the roots, stems, leaves, and grain dry matter accumulation of IM significantly increased by 32.42%, 34.52%, 11.78%, and 45.09%, respectively (2-year average, **Supplementary Figure S2A**). Compared with those of SP, the underground and aboveground dry matter accumulation of IP significantly decreased by 45.23%, 44.36%, 51.61%, and 60.25%, respectively (2-year average, **Supplementary Figure S2B**).

### 3.2 Changes in maize and peanut yield per plant and dry matter accumulation in planting box experiments

In the planting box experiments, compared with that of SM, the yield per plant of IM significantly increased by 16.4% (2-year average, **Table 2**). Compared with that of SP, the yield per plant of IP significantly decreased by 61.65% (2-year average, **Table 2**). The changes in dry matter accumulation of maize and peanut were consistent with those in the field experiment. Compared with those of SM, the roots, stems, leaves, and grain dry matter accumulation of IM significantly increased by 24.4%, 14.14%, 7.38%, and 20.14%, respectively (2-year average, **Supplementary Figure S3A**). Compared with those of SP, the roots, stems, leaves, and grain dry matter accumulation of IP significantly decreased by 28.95%, 47.84%, 31.19%, and 60.06%, respectively (2-year average, **Supplementary Figure S3B**).

**Table 2:** Changes in maize and peanut yields in a planting box experiment.

Planting patterns	2020	2021	2-year average
SM	304 ± 4.98*	270 ± 0.72**	287
IM	331 ± 2.34	355 ± 0.36	343
SP	22 ± 1.07**	21 ± 0.81**	21.5
IP	8.50 ± 0.90	8 ± 0.6	8.25

* and ** indicate significant differences between different planting patterns at *P*< 0.05 and *P*< 0.01, respectively.

SM, sole maize; SP, sole peanut; IM, intercropped maize; IP, intercropped peanut.

### 3.3 Interspecific interaction alters the spatial distribution of maize and peanut root length density

**Figure 2:**
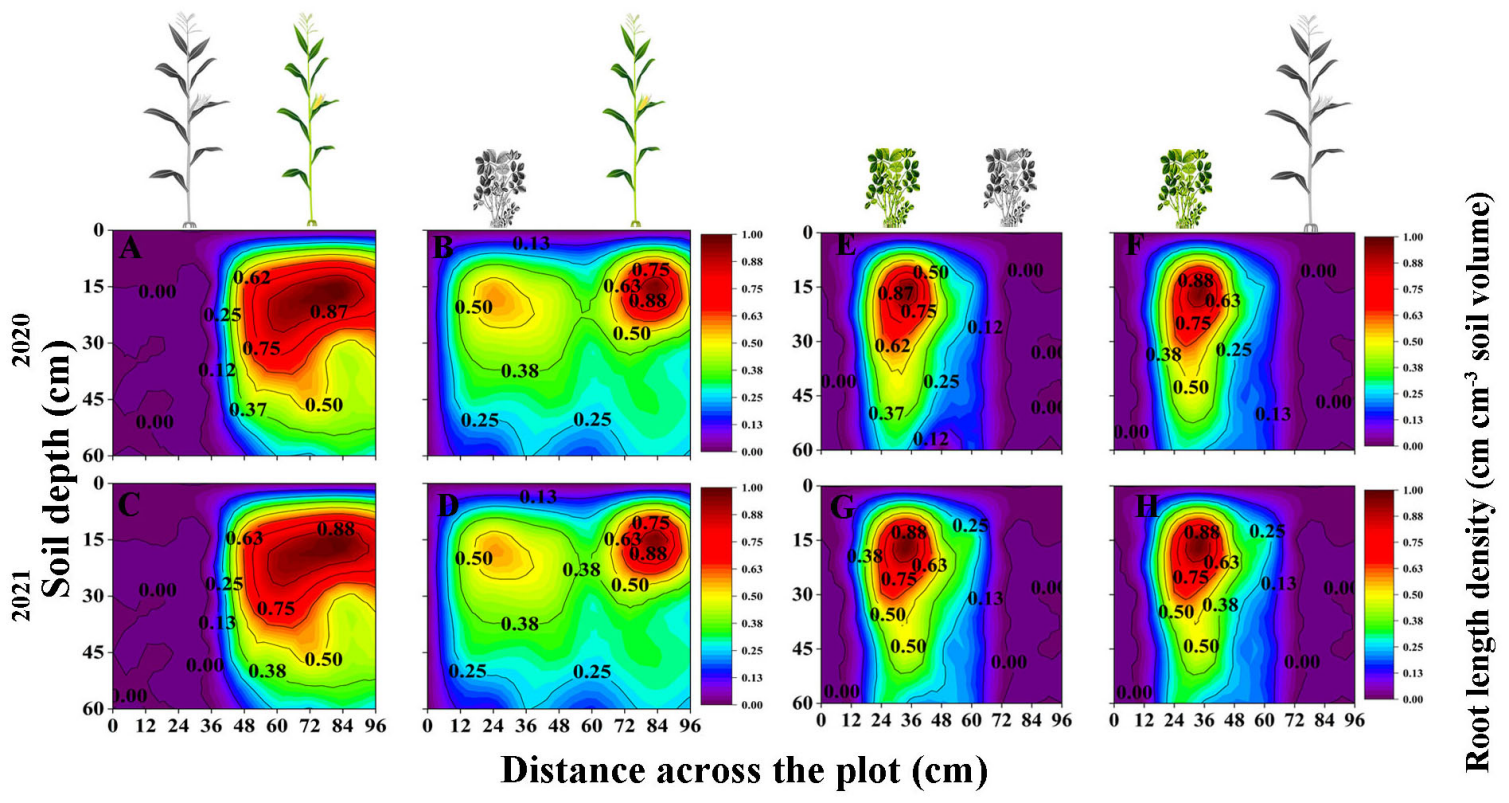
Spatial distribution of root length density (RLD, cm cm^-3^, based on Equation 5) of maize and peanut in different planting patterns. **(A, C)** sole maize (SM), **(B, D)** intercropped maize (IM), **(E, G)** sole peanut (SP), **(F, H) **intercropped peanut (IP), 0-36 cm: peanut inner row, 36-60 cm: interaction zone, 60-96 cm: maize inner row. The colour scale shows the normalized value of root length density (cm cm^-3^).

In general, the RLD of maize and peanut in the maize inner row, peanut inner row, and interaction zone significantly decreased with increasing soil depth. Compared with that of SM, the RLD of IM in the maize inner row increased by 69.01% and 42.67% at the 45–60 and 0–15 cm soil layers, respectively (2-year average, **Figures 2A-D**). The RLD of IM in the interspecies interactive zone decreased by 7.29% and 9.4% at the 0–15 and 15–30 cm soil layers and increased by 29.42% and 74.74% at the 30–45 and 45–60 cm soil layers, respectively (2-year average, **Figures 2A–D**). The RLD of IP was higher than that of SP at each soil depth, and the RLD in the peanut inner row increased by 1.65%, 1.76%, 3.86%, and 5.05%, respectively (2-year average, **Figures 2E–H**). The RLD of IP in the interspecific interaction zone decreased by 4.48%, 10.80%, 7.28%, and 5.66% with increasing soil depth (2-year average, **Figures 2E–H**).

### 3.4 Interspecific interaction alters the spatial distribution of maize and peanut root surface area density

**Figure 3:**
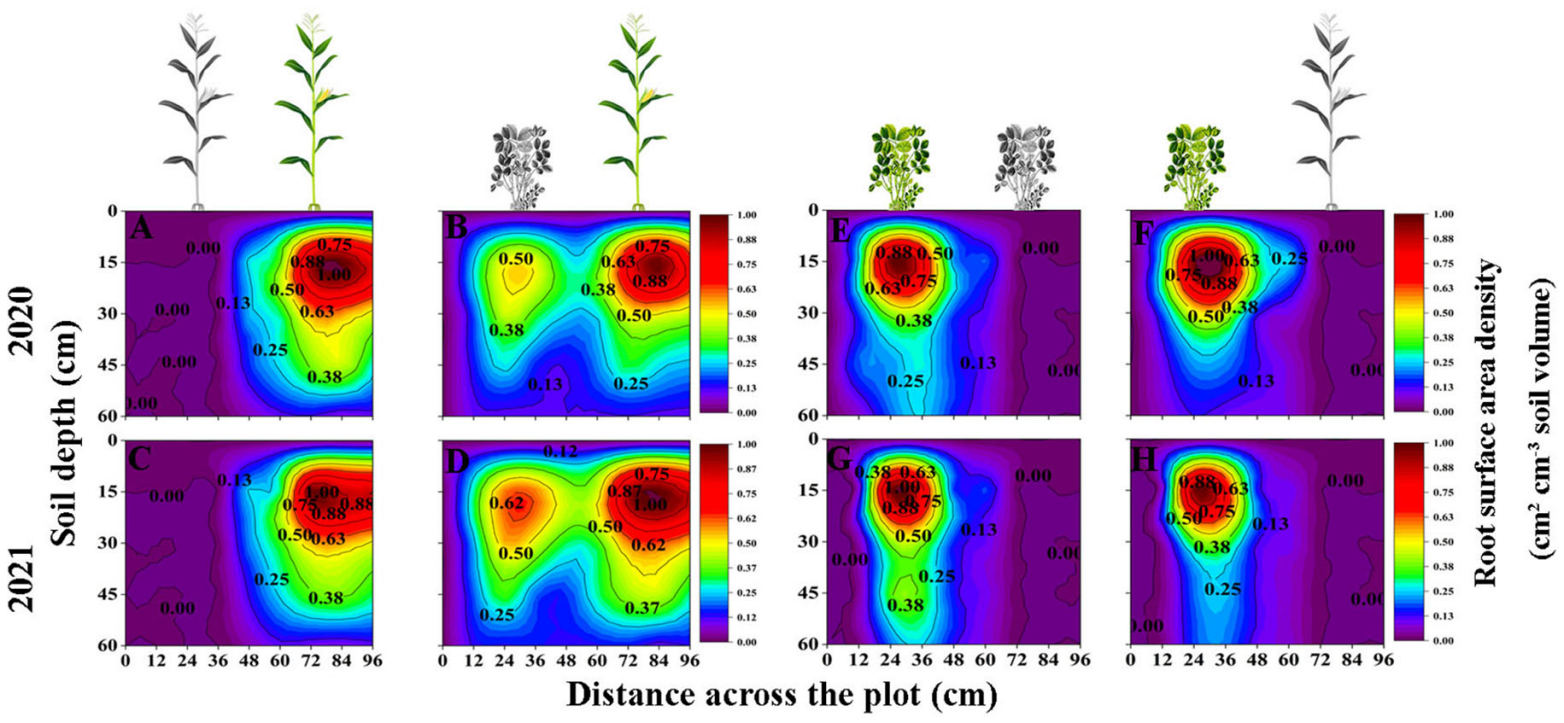
Spatial root surface area density (RSAD, cm^2^ cm ^-3^, based on Equation 6) distributions of maize and peanut in different planting patterns. **(A, C)** sole maize (SM), **(B, D)** intercropped maize (IM), **(E, G)** sole peanut (SP), **(F, H) **intercropped peanut (IP), 0-36 cm: peanut inner row, 36-60 cm: interaction zone, 60-96 cm: maize inner row. The colour scale shows the normalized value of root surface area density (cm^2 ^cm ^-3^).

The IM root system extended to the area planted with peanut and into deeper soil layers. The root distributions of SP and IP were limited to the space below the peanut plants (**Figure 3**). In general, the RSAD of maize and peanut in the maize inner row, peanut inner row, and interaction zone decreased significantly with increasing soil depth. Compared with that of SM, the RSAD of IM in the maize inner row increased by 10.3%, 13.71%, and 43.07% at 0–15, 15–30, and 45–60 cm, respectively (**Figures 3A, B**). The RSAD of IM in the interaction zone increased by 45.54% and 21.12% at 0–15 cm and 15–30 cm, respectively. Compared with that of SP, the RSAD of IP in the peanut inner row decreased by 12.15%, 12.55%, 42.48% and 38.70% at each soil depth, respectively. The RSAD of IP in the interaction zone decreased by 8.70%, 31.81%, 33.89%, and 33.92% at 0–15, 15–30, 30–45, and 45–60 cm, respectively (**Figures 3E, F**).

### 3.5 Interspecific interaction alters the spatial distribution of maize and peanut specific root length

**Figure 4:**
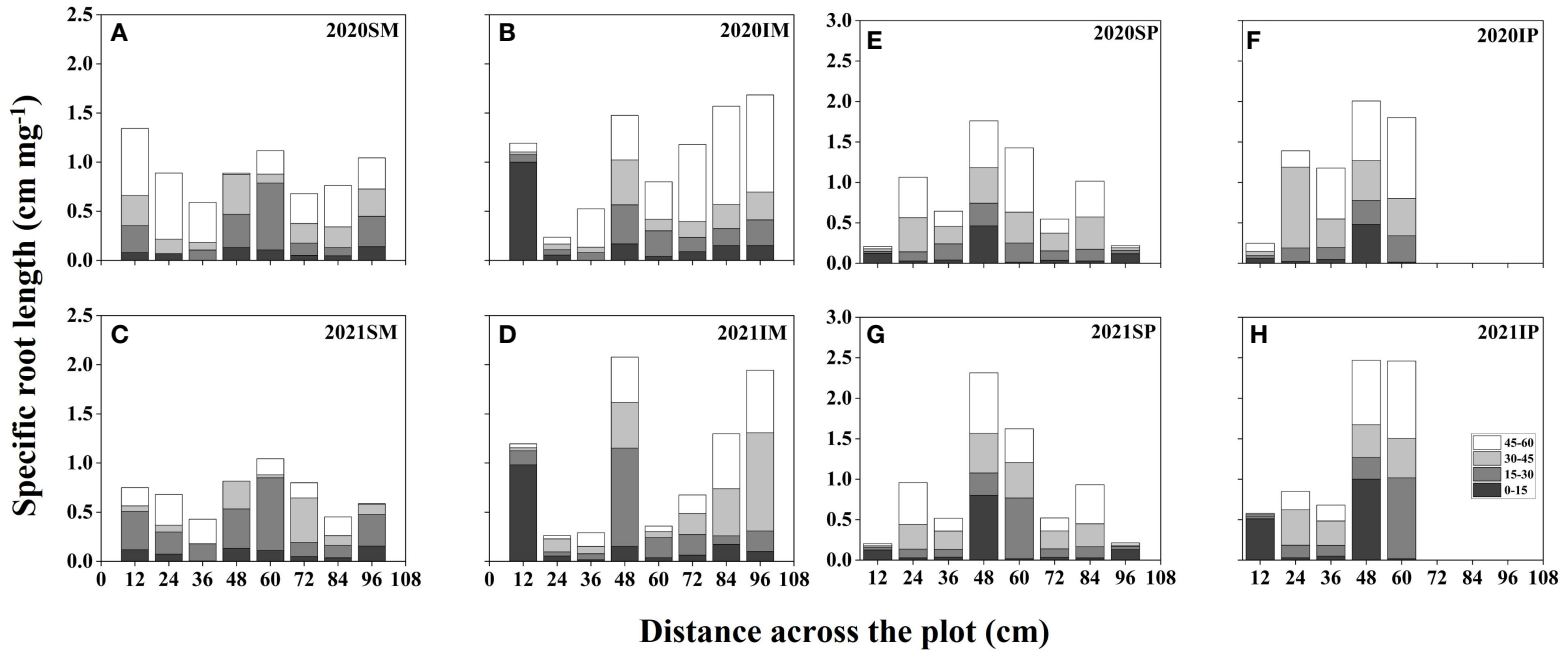
Specific root length (SRL, cm mg ^-1^, based on Equation 7) spatial distributions of maize and peanut in different planting patterns. **(A, C)** sole maize (SM), **(B, D)** intercropped maize (IM), **(E, G) **sole peanut (SP), **(F, H)** intercropped peanut (IP), SM, sole maize; IM, intercropped maize; SP, sole peanut; IP, intercropped peanut. 0-36 cm: peanut inner row, 36-60 cm: interaction zone, 60-96 cm: maize inner row.

**Figure 4** represents the spatial distributions of the SRL of maize and peanut under different planting patterns. In general, the SRL of maize and peanut in the maize inner row, peanut inner row, and interaction zone significantly increased with increasing soil depth. Compared with that of SM, the SRL of IM in the maize inner row increased by 22.59% and 113.06% at soil depths of 0–15 and 45–60 cm, respectively (2-year average, **Figures 4A–D**). In the interspecific interaction zone, the SRL of IM decreased by 9.32% and 12.24% at soil depths of 0–15 and 15–30 cm and increased by 16.91% and 80.65% at soil depths of 30–45 and 45–60 cm, respectively (2-year average, **Figures 4A–D**). Compared with that of SP, the SRL of IP in the peanut inner row increased by 90.28%, 19.91%, and 74.35% in the 0–15, 15–30, and 30–45 cm soil depths, respectively (2-year average, **Figures 4E–G**). In the interspecific interaction zone, the SRL of IP increased by 13.98%, 22.27%, 3.90%, and 37.77% at each soil depth (2-year average, **Figures 4E–G**).

### 3.6 Interspecific interaction alters the spatial distribution of maize and peanut root diameter

**Figure 5:**
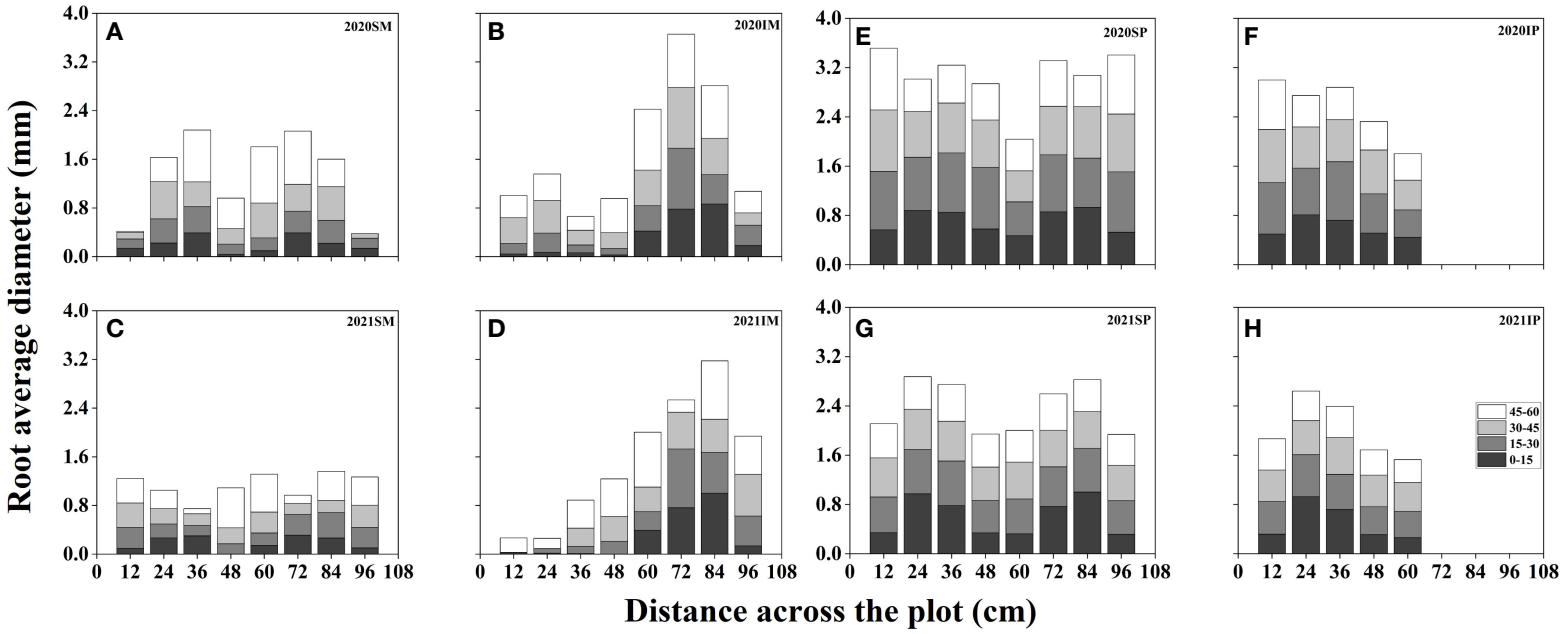
Spatial root diameter (RD, mm) distributions of maize and peanut in different planting patterns. **(A, C)** sole maize (SM), **(B, D)** intercropped maize (IM), **(E, G)** sole peanut (SP), **(F, H)** intercropped peanut (IP), SM, sole maize; IM, intercropped maize; SP, sole peanut; IP, intercropped peanut. 0-36 cm: peanut inner row, 36-60 cm: interaction zone, 60-96 cm: maize inner row.

**Figure 5** presents the spatial distribution of the RD of maize and peanut under different planting patterns. In general, the RD of maize and peanut in the maize inner row, peanut inner row, and interaction zone significantly decreased with increasing soil depth. Compared with that of SM, the RD of IM in the maize inner row increased by 100.91%, 52.15%, 41.48%, and 33.41% at soil depths of 0–15, 15–30, 30–45, and 45–60 cm, respectively (2-year average, **Figures 5A–D**). The RD of IM in the interaction zone increased by 66.09%, 14.91%, 7.32%, and 9.33% at soil depths of 0–15, 15–30, 30–45, and 45–60 cm, respectively (2-year average, **Figures 5A–D**). Compared with that of SP, the RD of IP in the peanut inner row decreased by 9.03%, 10.14%, 13.63%, and 12.63% at soil depths of 0–15, 15–30, 30–45, and 45–60 cm, respectively (2-year average, **Figures 5E, F**). In the interaction zone, the RD of IP decreased by 11.38%, 24.80%, 10.07%, and 22.32% at soil depths of 0–15, 15–30, 30–45, and 45–60 cm, respectively (2-year average, **Figures 5E, F**).

### 3.7 Correlation analysis of yield, dry matter accumulation, and root morphology

**Figure 6:**
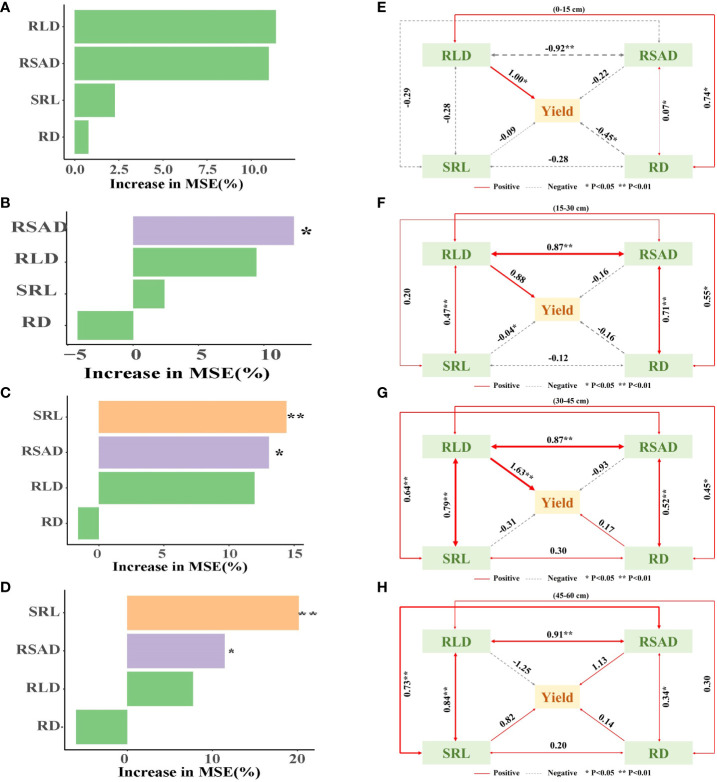
Maize and peanut root morphology indexes drive yield formation. **(A–D)** Relationships between yield per plant and root length density, root surface area density, specific root length and root diameter. **(E–H)** Structural equation model (SEM) of direct and indirect effects of the yield per plant, root length density, root surface area density, specific root length and root diameter. RLD, root length density; RSAD, root surface area density; SRL, specific root length; RD, root diameter. *0.01 < *p* ≤ 0.05, **0.001 < *p* ≤ 0.01.

Principal component analysis indicated that there was a positive relationship between maize RD and RSAD at a soil depth of 0–45 cm, between RLD and RSAD at a soil depth of 45–60 cm (**Supplementary Figures S4A–D**), and between peanut RD and RSAD at a soil depth of 0–60 cm (**Supplementary Figures S4E, F**). The Pearson correlation analysis revealed that maize RLD, RSAD, SRL, and RD were negatively correlated with maize yield per plant at soil depths of 0–60 cm (**Supplementary Figure S5A**). RLD, RSAD, SRL, and RD were positively correlated with peanut yield per plant at 0–60 cm soil depth, with RLD having the strongest correlation at 15–45 cm soil depth (**Supplementary Figure S5B**). A random forest model was used to assess the key factors affecting yield formation, and RSAD significantly affected yield at 15–60 cm. The SRL had a significant impact yield at 30–60 cm (**Figures 6A–D**). The results of structural equation modeling showed that root morphology indicators had a greater effect on yield with increasing soil depth, with a peak at 30–45 cm. The interaction between root morphology indicators was also more pronounced at 30–45 cm (**Figures 6E–G**).

## 4 Discussion

### 4.1 Yield and interspecific interactions in maize and peanut intercropping systems

Interactions between intercropping species contribute to intercropping yield advantages. Furthermore, dry matter accumulation is one of the important factors affecting yield (Zhang et al., 2022), which was demonstrated in this study. Similar results in the field experiment and planting box experiment demonstrated that the aboveground and underground dry matter accumulation of intercropped maize was significantly higher than that of sole maize, and the aboveground and underground dry matter accumulation of intercropped peanut was significantly lower than that of sole peanut (**Supplementary Figures S2, S3**). Importantly, the yield of intercropped maize was significantly higher than that of sole maize, and the yield of intercropped peanut was lower than that of sole peanut (**Tables 1**, **2**), consistent with the results of Li et al. (2019), indicating that the yield advantage of the intercropping system was mainly attributed to maize. This result supports objective 1 and can be explained by border-row effects, with the dominant species (maize) obtaining more resources in a maize–peanut intercropping system, which favors a yield advantage. Previous studies have shown that border row maize yields were on average 48% higher than middle rows, and border row peanut yields were on average 29% lower than middle rows (Wang et al., 2020). This is similar to the findings of this study, where changes in the proportion of border rows significantly affected the relative yield of the crop. This indicated that the intercropping system changes the light environment and promotes dry matter accumulation. Maize, as a C4 crop, not only is characterized by having high rates of photosynthesis but also achieves high levels of light interception and utilization efficiency because of height, which helps to promote sink and source capacities (Gou et al., 2017; Feng et al., 2020). It was observed in a maize alfalfa intercropping study that with a 75–134% increase in nitrogen fixation to give alfalfa the nitrogen it needed for growth, there was a 1.24–1.42-fold increase in nitrogen transfer to maize nitrogen (Shao et al., 2021). This is due to the interspecific competition that reduces mineral N in alfalfa rhizosphere soil and increases N fixation, which also supported complementary utilization. Thus, we speculated that in maize–peanut intercropping systems, maize, which has a side-row advantage, receives more light, nutrients, and resources than peanut (**Supplementary Figure S6**). These results suggested that maize yield was increased and yield advantages were achieved due to the border-row effects in the intercropping system.

The LER is one of the indicators used to assess land use efficiency in intercropping systems (Martin-Guay et al., 2018). We found that the LER was greater than 1 (**Table 1**). The results of the current study were consistent with the reported productivity of maize and peanut intercropping systems worldwide, i.e., the average LER was 1.31 (Feng et al., 2021). Maize and peanut intercropping has yield advantages and can lead to more efficient use of land resources than sole cropping; plant combinations of species, especially C3/C4 crop combinations, will greatly improve agricultural land use efficiency (Yu et al., 2015; Zhao et al., 2023a). The results were further validated by an analysis of the competitive abilities of the constituent crops. Highly competitive species have access to better ecological niches and gain more resources in intercropping systems, which is one of the reasons for the yield advantage in intercropping systems (Li et al., 2001; Bi et al., 2019; Wu et al., 2023). The *A* (0.94) and CR (1.54) values demonstrate the superior competitiveness of maize over peanuts in the intercropping system (**Table 1**). The results indicate that maize occupied superior ecological niches and had higher competitiveness than peanut. Conversely, because of the short stature of peanut, intercropping with maize led to a competitive disadvantage (Luo et al., 2021).

The morphological and spatial distribution of roots effectively reflects the ability of crops to compete for nutrients and water in intercropping systems (Ma et al., 2019; Zhao et al., 2022b). The results supported the third objective, that is, the spatial distribution of root length density (**Figure 2**), root surface area density (**Figure 3**), and root diameter (**Figure 5**) promoted the competitiveness of maize, and the spatial distributions of root surface area density and root diameter were suppressed in peanut, which was a weak competitor. Previous studies have shown that in maize–soybean intercropping systems, the root length of intercropped maize and the root length density of intercropped maize increased by 17.97%–44%, while the root length density of intercropped soybean decreased by 30.69%–46.46% (Wei et al., 2022). Maize showed greater root morphological plasticity than legumes, and the changes in root length density, root weight density, and total root surface area in an intercropping system were greater than those in sole cropping (Yang et al., 2022). Consequently, the spatial distribution characteristics of root morphological indexes in response to interspecific interactions can explain the phenomenon of high yields in maize–peanut intercropping (Zhang et al., 2023). This result answers our aim 3, that the dominant species, maize, had higher root length density and root surface area density than peanut in the intercropping system. Differences in the response of root morphological indicators to interspecific effects are a factor in the yield advantage of intercropped maize.

In this study, intercropped maize in the maize inner row had higher RLD, RSAD, and RD than sole maize at each soil depth (**Figures 2**–**5**). Compared with sole peanut, intercropped peanut in the peanut inner row had higher RSAD and RD at each soil depth (**Figures 3**, **5**). In addition, the intercropped maize root system extended into the peanut row and below the space occupied by peanut. Additionally, RLD, RSAD, and RD were higher in intercropped maize than in sole maize in the interspecific interaction zone, while RSAD and RD were lower in intercropped peanut than in sole peanut. This result indicated that maize had a competitive advantage in the intercropping system, with optimized root distribution, expanded nutrient absorption area, and increased nutrient acquisition efficiency, resulting in the maximization of the utilization rate of soil resources (Fort et al., 2014; Oram et al., 2018). Thus, well-developed fine roots and an optimized root distribution promote nutrient uptake by crops (Zheng et al., 2022).

Previous studies have shown that temporal or spatial niche differentiation of species influences interspecific competition and resource access (Zhang et al., 2015; Homulle et al., 2021). In the overlapping part of the root system, that is, the interspecific interaction zone, the increase in RLD, RSAD, SRL, and RD of intercropped maize was greater than that of intercropped peanut (**Figures 2**–**5**), indicating that maize showed greater root morphological plasticity than peanut. The strong competitiveness of maize optimizes the distribution of root morphology and enhances its ability to compete with neighboring peanuts for water and nutrients (Christina et al., 2023; Wei et al., 2024). Moreover, this explains the decrease in the RSAD and RD of intercropped peanut. Since the root distribution of intercropped maize extends below the peanut plants, occupying a larger soil space, the lateral distribution of peanut roots is reduced. Thus, differences in root morphology between intercropped maize and intercropped peanut lead to less niche overlap and drive positive complementary effects (Yu et al., 2022; Zhao et al., 2022b).

### 4.2 The correlation between yield and the spatial distribution of root morphology

In this study, the PCA results showed a positive correlation between RLD and RSAD at soil depths of 45–60 cm (**Supplementary Figure S4**), suggesting an interaction that could impact yield (Gao et al., 2024). Pearson’s analysis showed that the effect of root morphological indicators on crop yield varied in soils of different depths (**Supplementary Figure S5**). The effects of RLD and RSAD on crop yield were gradually revealed with increasing soil depth. In particular, the effect of RSAD on yield has been significant in the 15–60 cm depth range, while SRL also had a significant effect on yield at 30–60 cm. This suggested that there was a positive correlation between root morphological indicators and yield in certain soil depth ranges. For example, Zhang et al. (2014) demonstrated that root length density and root diameter decreased and specific root length increased in intercropped walnut and intercropped wheat compared to sole walnut and sole wheat due to underground root competition, which resulted in lower yields and biomass for both crops. This provides a new perspective for a deeper understanding of the relationship between root characteristics and crop yield at different soil levels. Moreover, structural equation modeling showed that the correlation of root morphology indicators with yield increased progressively as soil depth increased, reaching its peak at 30–45 cm (**Figure 6**). Meanwhile, the interaction between root morphology indicators was also more pronounced in the 30-45 cm soil layer, which may have a synergistic effect on yield. These results suggest that the spatial distributions of maize and peanut roots in intercropping systems show great plasticity and importance in regulating maize and peanut productivity in response to interspecific competition. This is also consistent with recent research reports (Yang et al., 2022; Zhang et al., 2023) that plasticity and differences in crop root traits were partly responsible for overproduction in maize–legume intercropping systems. The characteristics of root distribution across varying soil depths to strategically implement cultivation practices, with the goal of enhancing the benefits of root spatial distribution and ultimately increasing crop yield and quality (Dhima et al., 2007). By conducting a comprehensive analysis of the correlation between root morphology and yield, this research can offer a scholarly foundation for agricultural practices and facilitate enhancements in crop yield and production efficiency.

## 5 Conclusion

Maize–peanut intercropping improves yields and land use efficiency through border-row effects. Compared with that of sole maize, the yield of intercropped maize significantly increased, while the yield of intercropped peanut significantly decreased compared with that of sole peanut. Additionally, intercropped maize showed higher *A* (0.94) and CR (1.55), indicating that maize is the dominant species and has a greater competitive ability than peanut. This also indicates that the yield advantage was mainly due to maize border-row effects. Furthermore, the spatial distribution of intercropped maize roots extended below the space occupied by peanuts, especially in the interspecific interaction zone, where the RLD, RSAD, and RD at each soil depth were higher for intercropped maize than for sole maize and the RLD and RSAD of intercropped peanut were lower than those of sole peanut. It is clear that maize exhibits greater root morphological plasticity than peanut, expanding the nutrient uptake area and thus achieving interspecific complementary effects in intercropping systems. Hence, it is essential to understand the responses of root morphology and spatial distribution plasticity to border-row effects in maize–peanut intercropping systems to achieve sustainable agricultural development and efficient use of limited resources.

## Data availability statement

The original contributions presented in the study are included in the article/**Supplementary Material**. Further inquiries can be directed to the corresponding authors.

## Author contributions

QD: Data curation, Formal analysis, Writing – original draft. XZ: Funding acquisition, Supervision, Conceptualization, Visualization, Writing – review & editing. YS: Methodology, Project administration, Validation, Writing – review & editing. DZ: Methodology, Project administration, Writing – review & editing. GL: Project administration, Software, Writing – review & editing. JP: Methodology, Project administration, Writing – review & editing. CF: Supervision, Validation, Visualization, Writing – review & editing. HZ: Supervision, Validation, Visualization, Writing – review & editing. XS: Software, Supervision, Writing – review & editing. XL: Supervision, Validation, Writing – review & editing. JZ: Validation, Visualization, Writing – review & editing. ZS: Supervision, Validation, Visualization, Writing – review & editing. HY: Funding acquisition, Supervision, Writing – review & editing.
