# Supplementary material for: Border row effects improved the spatial distributions of maize and peanut roots in an intercropping system, associated with improved yield
Source: Front Plant Sci. 2024 Jun 26;15:1414844. doi: 10.3389/fpls.2024.1414844 (PMC11233825; doi:10.3389/fpls.2024.1414844)
Supplement: Supplementary file 1 [file DataSheet_1.docx]

Supplementary Material

# Supplementary Figures and Tables

## Supplementary Tables

**Supplementary Table 1 Fertilization amount of maize and peanut (kg·hm^-2^)**

| **Peanut** | | |  | **Maize** | | |
| --- | --- | --- | --- | --- | --- | --- |
| **N** | **P_2_O_5_** | **K_2_O** |  | **N** | **P_2_O_5_** | **K_2_O** |
| 180 | 85 | 95 |  | 89.96 | 599.70 | 119.94 |

## Supplementary Figures


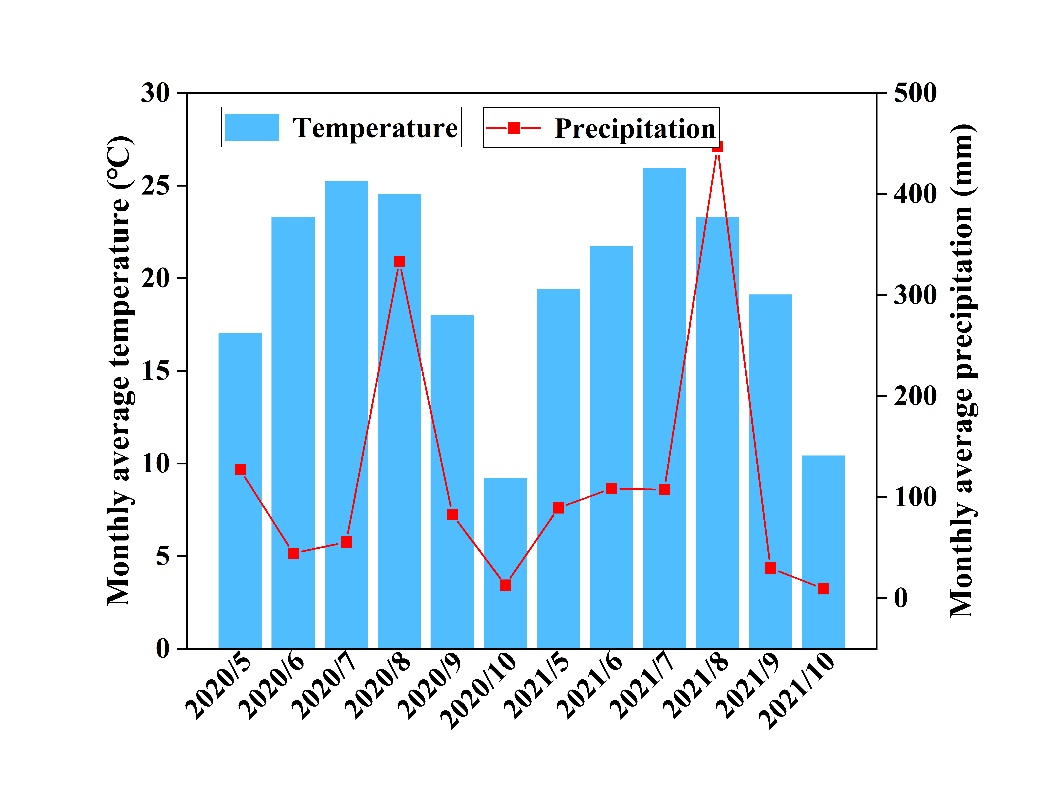


**Supplementary Figure 1** Average temperature and precipitation for the 2020-2021 growing season.


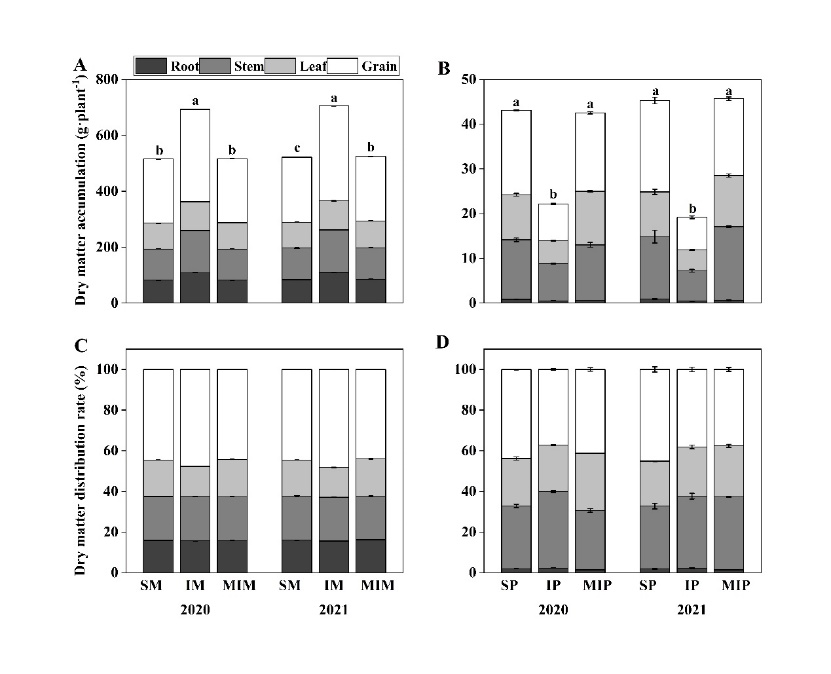


**Supplementary Figure 2** Maize and peanut dry matter accumulation in field experiment from 2020-2021. Different lowercase letters indicate dry matter accumulation per plant significant differences between different planting patterns at P < 0.05. SM: sole maize, IM: intercropped maize, MIM: the middle row of intercropped maize, SP: sole peanut, IP: intercropped peanut, MIP: the middle row of intercropped peanut.


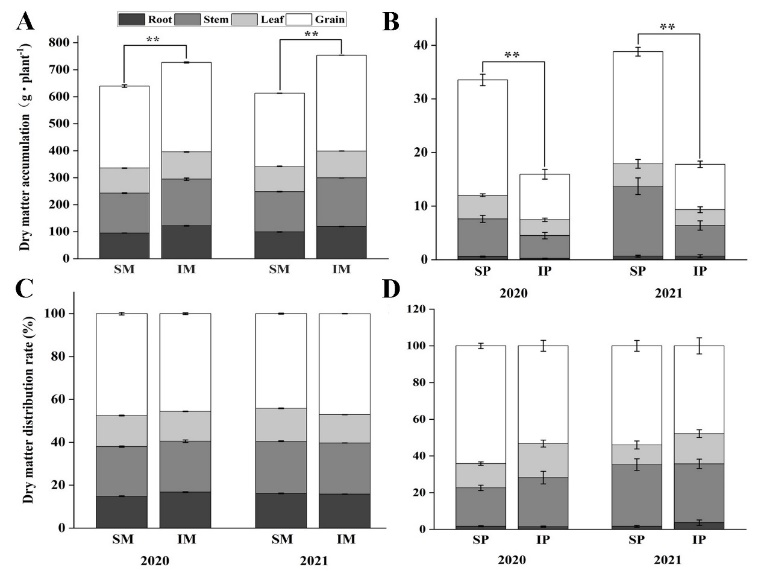


**Supplementary Figure 3** Maize and peanut dry matter accumulation in planting box experiment from 2020-2021. Different lowercase letters indicate dry matter accumulation per plant significant differences between different planting patterns at P < 0.05. SM: sole maize, IM: intercropped maize, SP: sole peanut, IP: intercropped peanut.


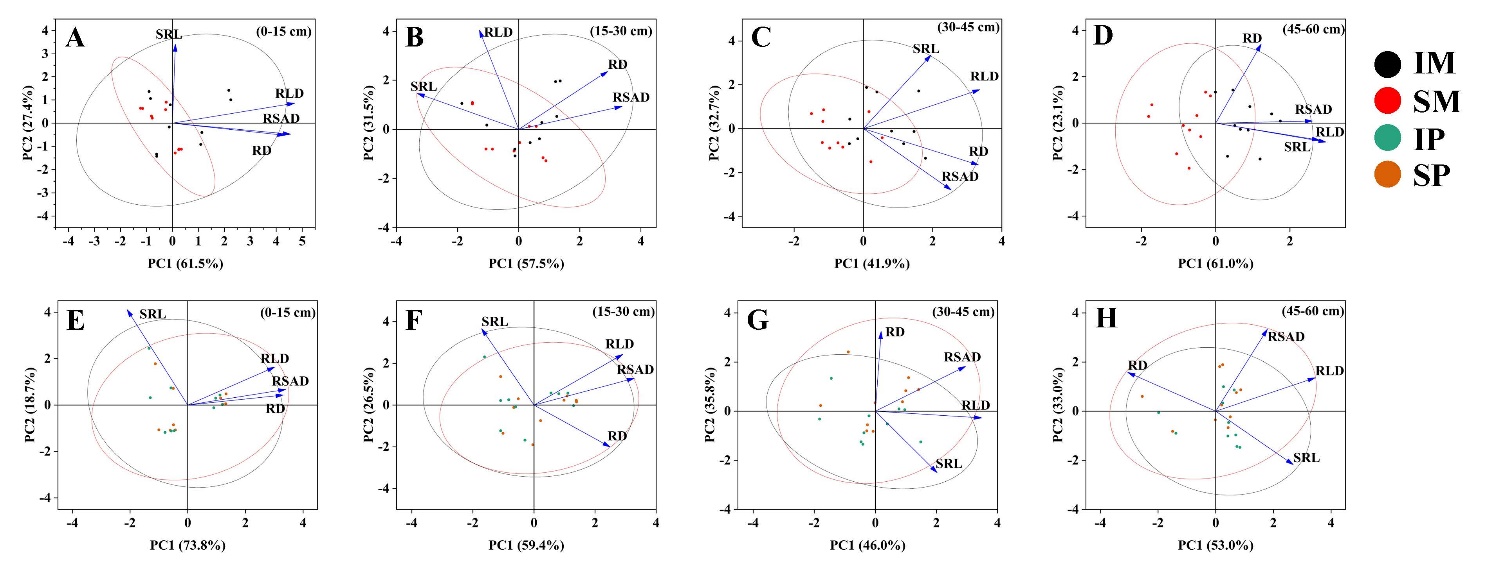


**Supplementary Figure 4** Principal component analysis (PCA) of normalized root morphology for maize and peanut in different soil depths. The percentages represent the proportion of variation for which the axis accounts. (A-D), Root morphology for maize at 0-15 cm, 15-30 cm, 30-45 cm, 45-60 cm, respectively. (E-F), Root morphology for peanut at 0-15 cm, 15-30 cm, 30-45 cm, 45-60 cm, respectively. SM: sole maize, IM: intercropped maize, SP: sole peanut, IP: intercropped peanut.


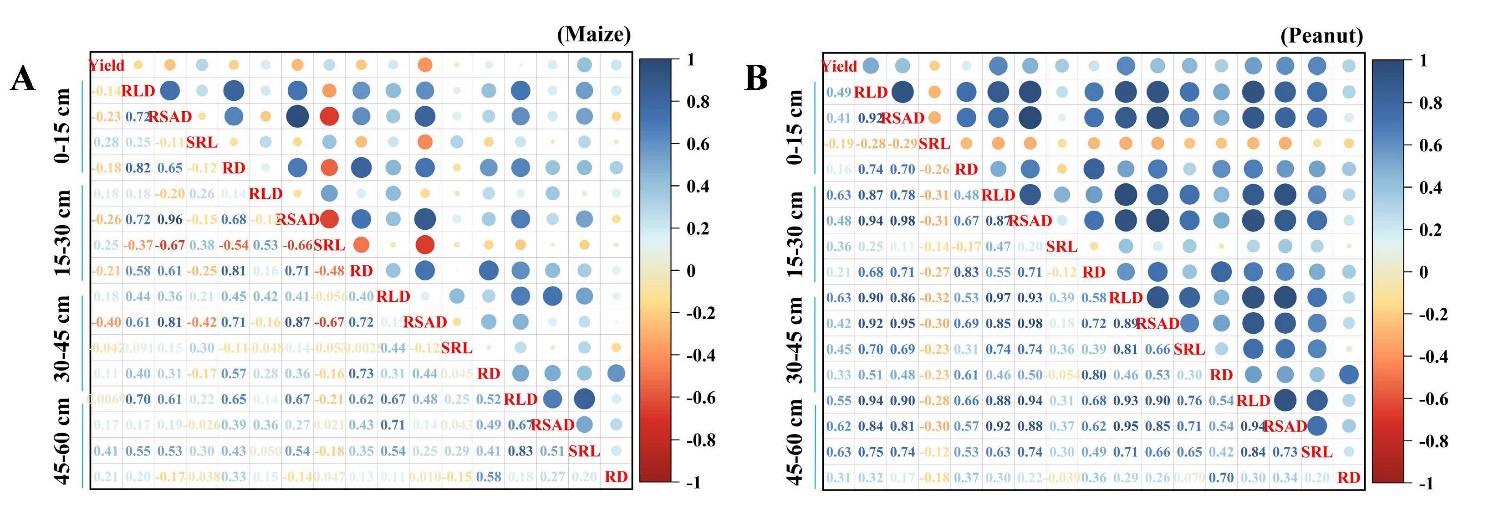


**Supplementary Figure 5** Pearson correlation matrix of potential relationships between yield per plant and root morphology of maize (A) and peanut (B). Values were considered significant at P<0.05. The circles and values in blue and red represent positive and negative correlations, respectively. RLD (0-15), (15-30), (30-45), and (45-60): root length density at soil depths of 0-15 cm, 15-30 cm, 30-45 cm, and 45-60 cm, respectively. RSAD (0-15), (15-30), (30-45), and (45-60): root surface area density at soil depths of 0-15 cm, 15-30 cm, 30-45 cm, and 45-60 cm, respectively. SRL (0-15), (15-30), (30-45), and (45-60): specific root length at soil depths of 0-15 cm, 15-30 cm, 30-45 cm, and 45-60 cm soil depth, respectively. RD (0-15), (15-30), (30-45), and (45-60): root diameter at soil depths of 0-15 cm, 15-30 cm, 30-45 cm, and 45-60 cm soil depth, respectively.


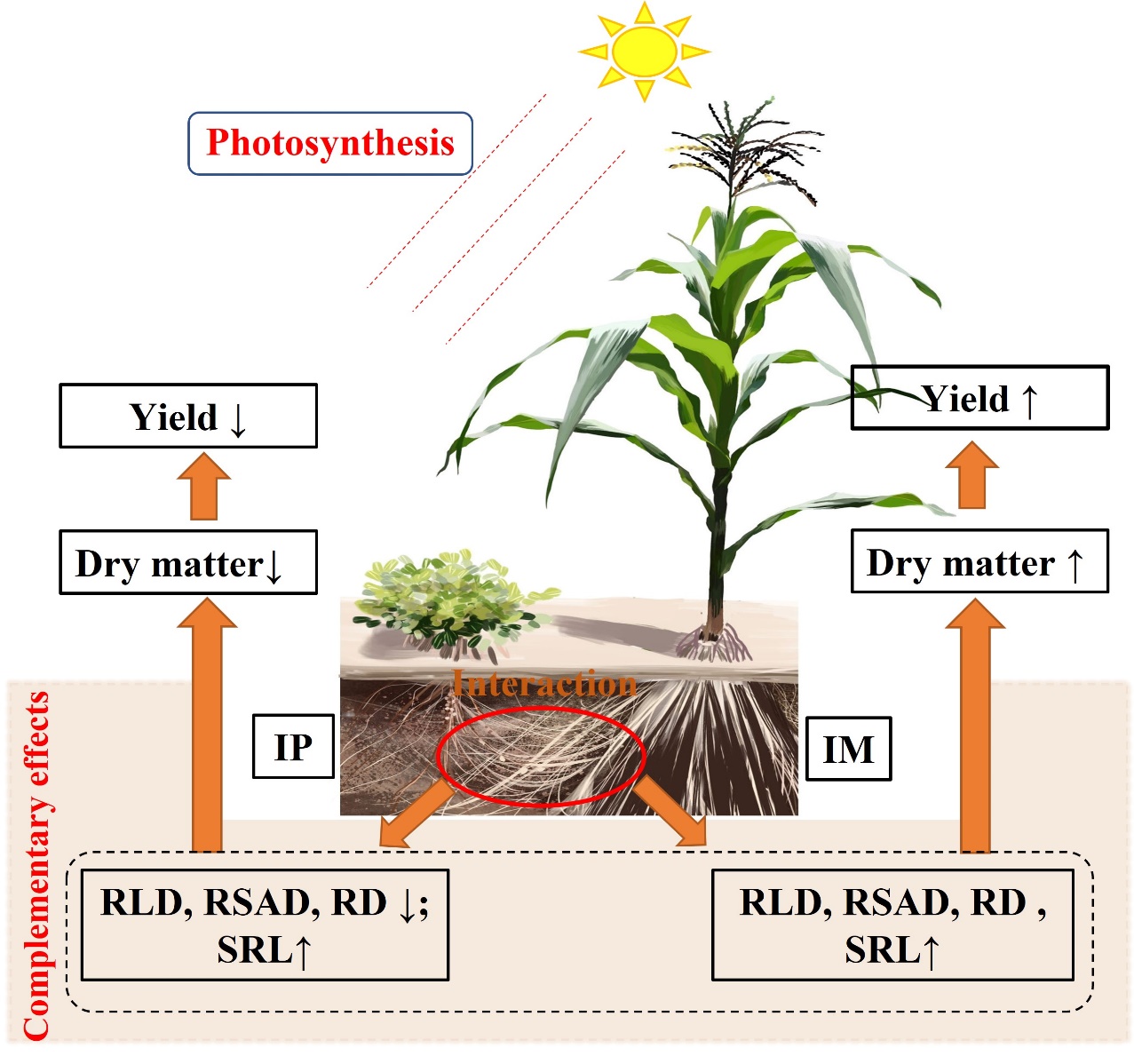


**Supplementary Figure 6** Simulation of root interactions mediating changes in root morphology for yield improvement.
